# Risk Factors Associated With Outbreaks of Infectious Salmon Anemia (ISA) With Unknown Source of Infection in Norway

**DOI:** 10.3389/fvets.2018.00308

**Published:** 2018-12-06

**Authors:** Trude Marie Lyngstad, Lars Qviller, Hilde Sindre, Edgar Brun, Anja B. Kristoffersen

**Affiliations:** Norwegian Veterinary Institute, Oslo, Norway

**Keywords:** infectious salmon anemia, ISA, ISAV, HPR0, HPR-del, Atlantic salmon, risk factors

## Abstract

The occurrence of infectious salmon anemia (ISA) outbreaks in marine farmed Atlantic salmon constitutes a recurring challenge in Norway. Here, we aim to identify risk factors associated with ISA outbreaks with an unknown source of infection (referred to as primary ISA outbreaks). Primary ISA outbreaks are here defined by an earlier published transmission model. We explored a wide range of possible risk factors with logistic regression analysis, trying to explain occurrence of primary ISA with available data from all Norwegian farm sites from 2004 to June 2017. Explanatory variables included site latitude and a range of production and disease data. The mean annual risk of having a primary outbreak of ISA in Norway was 0.7% during this study period. We identified the occurrence of infectious pancreatic necrosis (IPN), having a stocking period longer than 2 months, having the site located at high latitude and high fish density (biomass per cage volume) in the first six months after transfer to sea site as significant risk factors (*p* < 0.05). We have identified factors related to management routines, other disease problems, and latitude that may help to understand the hitherto unidentified drivers behind the emergence of primary ISA outbreaks. Based on our findings, we also provide management advice that may reduce the incidence of primary ISA outbreaks.

## Introduction

Infectious salmon anemia (ISA) is an infectious disease that causes high mortality in farmed Atlantic salmon (1), with severe implications for production economics. The disease is listed by the Office International des Epizooties (OIE, www.oie.int) and the European Commission (Council Directive 2006/88/EC). Infectious salmon anemia is caused by infection with the ISA virus (ISAV), which is from the *Orthomyxoviridae* family. Since being first recorded in 1984 in Norway, ISA outbreaks have been reported in farmed Atlantic salmon in countries such as Scotland, the United Kingdom, Canada, USA, the Faroe Islands, and Chile (2).

Infectious salmon anemia was a serious disease problem in salmon aquaculture in Norway during the late 80s and early 90s, with at least 80 new outbreaks in 1990 at the peak of the epidemic. A number of biosecurity measures were implemented to control the horizontal spread of ISA in the early 90s (3). Although there was a substantial decrease in the number of outbreaks, ISA was not eradicated and remained a disease problem. Since 1993, the annual number of ISA outbreaks has varied between 1 and 20. In addition to the animal welfare concerns, each outbreak induces high rates of mortality, which are costly (1), as well as management restrictions that limit production in the surrounding salmon production sites (4).

There are two main variants of ISAV, one variant is highly virulent and associated with ISA outbreaks, termed HPR-deleted ISAV (HPR-del ISAV). The other variant (termed HPR0 ISAV) is assumed to be non-virulent. HPR0 ISAV was proposed in 2002 to be an ancestral form of HPR-del ISAV (5). A direct link between HPR0 ISAV and HPR-del ISAV remains to be demonstrated, but a strong indication of the evolution from HPR0 ISAV to HPR-del ISAV was recently reported from a Faroese Atlantic salmon marine farm (6). HPR0 ISAV is prevalent and described as a transient infection (7–10). Interestingly, a high proportion of fish groups experience infection with HPR0 ISAV during the seawater production phase without evolving into the virulent HPR-del ISAV (9–11). This shows that HPR0 ISAV may represent a risk for the emergence of clinical ISA, even though the risk of transition from HPR0 ISAV to HPR-del ISAV appears to be low.

Horizontal transmission is an important mechanism in the spread of ISA, and it explains local epidemics well (12–15). According to a previously described transmission model for ISAV, nearly half (43%) of the outbreaks in Norway from 2004 to 2009 were explained by the spread between proximate farms (12). The model uses both geographical distances and genetic similarities between virus isolate sequences to identify the source of infection and to establish transmission networks between farms. The model could not explain the source of infection for the remaining 57%, and these cases were considered as either the first outbreak in local epidemics or as a single event in cases with no further spread. Outbreaks with unknown sources of infection were therefore defined as primary ISA outbreaks.

Vertical transmission of ISAV has been suggested to occur and could explain the emergence of primary ISA outbreaks (16–18). Transmission over generations might occur due to contamination during the stripping process if not by true vertical transmission. Experiences from the Faroe Islands suggest that transmission of HPR0 ISAV between juvenile salmon and marine salmon, and between marine salmon and brood fish, may occur. These authors have not been able to demonstrate a similar link between brood fish and juvenile salmon (19). Knowledge of the possible impact of a marine reservoir on the emergence of ISA is still scarce. The ISAV receptor is present in a range of fish species (20). Farmed salmon are exposed to a large range of wild fish/reservoirs in the surrounding environment during the seawater production phase because salmon farming in Norway takes place in open cages in the sea. All these transmission pathways could contribute to primary ISA outbreaks.

The transmission mechanisms behind primary ISA outbreaks depend on the introduction of either high virulent strains from unknown reservoirs or non-pathogenic strains of the ISA virus (HPR0 ISAV) that facilitate an evolution toward higher virulence. Therefore, an increased susceptibility of a salmon production site may introduce risk of a primary ISA outbreak. In addition, factors like viral reproduction and shedding may affect the rate of evolution in the virus. Suboptimal management, stocking and fallowing routines, disease events, production density (number of fish on farm), handling and treatment of fish are all factors that have been associated with an increased risk of ISA outbreaks (21–24). Good management and biosecurity as defined by the Office International des Epizooties (OIE) (25), are therefore in general considered important for the prevention and control of ISA, but these factors have not been structurally examined specifically for primary cases. We argue that a better understanding of factors associated with an increased risk of primary ISA outbreaks may elucidate unknown transmission mechanisms and may help to optimize preventive measures, facilitate early detection, and reduce the risk of local ISA epidemics.

Here, we aim to identify potential risk factors that are more associated with marine sites and fish cohorts contracting primary ISA outbreaks, as defined by a previously published transmission model (12), than those that do not. We further aim to evaluate their role as potential drivers behind such outbreaks in farmed Atlantic salmon in Norway.

## Materials and Methods

### Data Preparation and Definitions

This study was based on salmon production statistics, geographical information and disease data from 2004 to June 2017 in Norway. We defined the study units as fish groups, which are cohorts of seawater-reared Atlantic salmon stocked on the same site and separated by a fallowing period. Only fish groups that were kept at one specific site for at least 6 months were included in the study. Geographical references for sites and slaughterhouses were obtained from the aquaculture register of the Directorate of Fisheries (*www.fiskeridir.no*). Monthly site reports on production statistics to the Norwegian authorities on biomass data, including number of fish, weight and volume and seawater temperature, were obtained as described in Kristoffersen et al. (26). Disease data on ISA, infectious pancreatic necrosis (IPN), heart and skeletal muscle inflammation (HSMI), and pancreas disease (PD) were compiled from the Norwegian Veterinary Institute (NVI).

**Figure 1:**
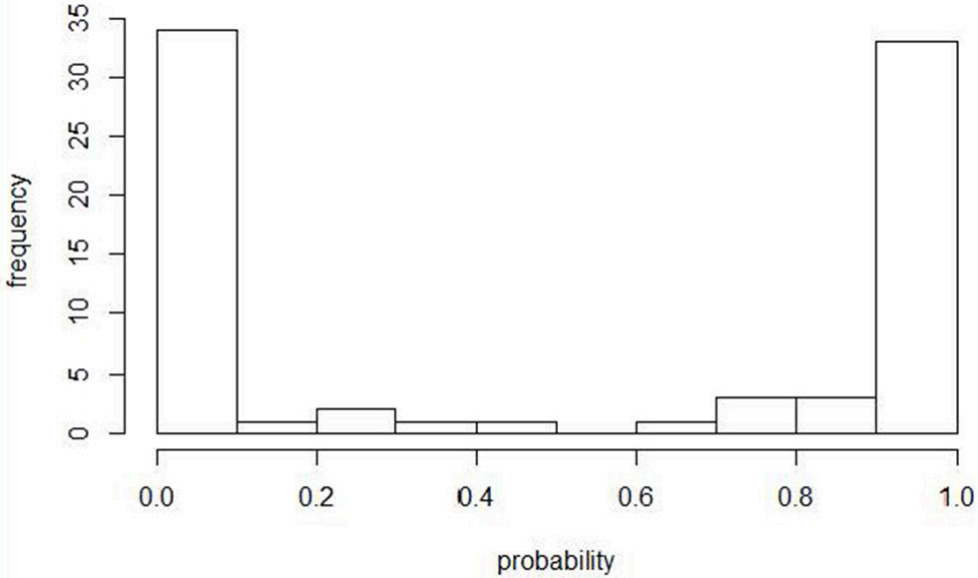
Histogram showing the estimated probabilities for being infected by neighboring farms after a rerun of the model defining primary outbreaks (12). The figure includes 80 fish groups with confirmed ISA during the study period 2004–June 2017. Fish groups with an estimated probability <10% were defined as primary ISA outbreaks.

An ISA outbreak was defined according to the contingency plan for the control of ISA in Norway (4), which is in accordance with the criteria described by the OIE (2). Predictions from the previously described model by Aldrin et al. (12) were re-run with ISA outbreak data corresponding to our study period. The probability distribution of fish groups being infected by a neighboring farm is shown in Figure 1. As our focus was primary ISA outbreaks, we defined the cases as being fish groups with an ISA outbreak and with an estimated probability of <10% for being infected by a neighboring farm (i.e., primary ISA outbreak). The cut-off of 10 % was chosen similarly as in Lyngstad et al. (10), implying a high probability of an unknown source of infection in these outbreaks. Two ISA outbreaks were omitted from the list of primary ISA outbreaks (Outbreak no 201407 and 201605) because epidemiological/field information from the Norwegian Food Safety Authority (NFSA) indicated that these outbreaks could be explained by contact to previous ISA sites (i.e., high probability of horizontal transmission). One ISA outbreak (Outbreak no. 201601) was added to the case list because epidemiological/field information from NFSA indicated that this outbreak was most likely a primary ISA outbreak. Outbreaks with a known source of infection were not defined as controls, and because they were not susceptible to ISA during the entire grow-out period they were removed from the dataset to avoid bias. Controls were defined as any other fish group in the study period regardless of health status and geographical location along the coast. The controls were not tested for ISA, but a sensitivity analysis of the model was done by excluding controls highly exposed to ISAV from neighboring farms regardless of ISA status.

Using a similar approach as that described in Bang Jensen and Kristoffersen (27), we used data on salmon production, geographical coordinates and disease status to generate explanatory variables. In order to have comparable data between cases and controls, we chose to extract variables related to the production statistics (number of fish, weight and volume) and temperature during the first 6 months after transfer to a sea site.

The following risk indicator variables were included in the analysis:

- Latitude: The south-north geographic reference for the farm site of the fish groups in a geographic coordinate system, acquired from the aquaculture register of the Directorate of Fisheries.
- Distances: Euclidean distances from farm site to coastline and seaway distance from the farm site to the nearest slaughter house.
- Temperature: Number of months below a given sea temperature threshold. The optimal threshold was found by testing all integer temperatures between 4 and 13 degrees Celsius.
- Fallowing: Number of months a sea site has been empty before restocking.
- Site index: in order to explore whether an intensively used site was at higher risk of contracting a primary ISA outbreak, we calculated an index per farm site as the sum of active months per fish group divided by the total number of months, encompassing the time from when the first fish group was put to sea until the last fish group on site was slaughtered.
- Transfer to sea: The year when a fish group was transferred to the sea site.
- Cohort type: Fish groups were categorized into “spring” cohorts if the fish were put to sea between March and July, “autumn” if the fish were put to sea between August and February, or “relocated” if the fish start weight was higher than 250 grams.
- Stocking duration: Number of months until the maximum number of fish on a site was reached (i.e., to complete the group), categorized into “normal” (1–2 months) or “long” (more than 2 months).
- Weight at sea transfer: mean weight of smolt (gram).
- Max number of fish: Maximum number of fish at site at any time the first 6 months.
- Max density: Maximum biomass density calculated as maximum biomass per cage volume at any time the first 6 months.
- Mortality: Mean and maximum mortality during the first 6 months.
- The area production density (APD): Calculated for each fish group and site as the monthly kernel density of average monthly biomass on the site and the surrounding sites within 40 km, following Jansen et al. (28). For the logistic regression analyses, we included maximum and mean values for APD for (1) the fish group and surrounding sites and for (2) surrounding sites of a fish group only. Results for mean APD values were not reported.
- Other diseases: The occurrence after transfer to sea of heart and skeletal muscle inflammation (HSMI), pancreas disease (PD) or infectious pancreatic necrosis (IPN) in fish groups, either before a primary ISA outbreak (cases) or anytime during the seawater production period (controls), was compiled from the list of reported cases. PD has been notifiable since 2007 at a national level and NVI, as the reference laboratory for PD, performed all diagnostics prior to 2007. The list should therefore be exhaustive (29). IPN was notifiable up to 2008 and HSMI between 2008 and 2013, and reporting may thus not be exhaustive. However, reporting and investigating abnormal mortalities have always been mandatory, and we therefore assume that the list include most clinical cases of IPN and HSMI. None of the fish groups identified as cases had PD before a primary ISA outbreak (data not shown).

### Statistical Analyses

Multivariable logistic regression [glm, library mgcv in R (30)] was performed, and all explanatory variables were tested separately as predictors in univariable logistic regression. Temperature was tested as univariable for a range of temperature thresholds, and the threshold that gave the best univariable fit to the response was used in the future analyses. Variables having *p* < 0.25 in the univariable analysis were included in a multivariable regression model selection. All continuous explanatory variables were explored with and without log transformation in the univariable regression models to find the optimal fit and approximate the normal probability distribution. The multivariable modeling was performed using a backward selection process, subtracting one variable at a time from a model that included all covariates, and correlated covariates with an *r*^2^ > 0.7 were not included in the same model. The simplest model among model alternatives with the lowest AIC values was selected as the final model. Interactions between all explanatory variables in the final model were tested. Residual patterns from the top-ranked model indicated that random effects with site or company as grouping variables were not applicable (not reported). The estimates and *p*-values for the final model were reported together with odds ratio for the discrete variables. The final model was used to predict the probability of a primary ISA outbreak for four different scenarios at four different latitudes.

In our data, two of the sites had two occurrences of primary ISA outbreaks. We assessed the probability for this outcome using a permutation test: 33 fish groups (= number of primary ISA outbreaks in the data set) were drawn at random from our study population to simulate primary ISA outbreaks occurring purely by chance. This was repeated 10,000 times, and the percentage of the samples that included two or more duplicated sites was reported.

Data management and analyses were performed using R version 3.4.1. (31). The maps in **Figures 3, 4** were made using ArcGIS software.

## Results

**Figure 2:**
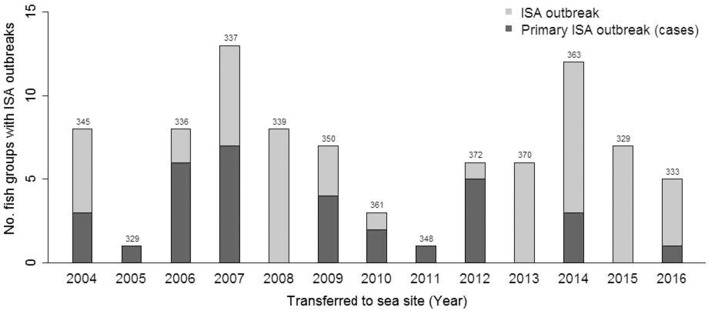
Bar plot showing number of fish groups transferred to sea each year that obtain ISA during the production period. The dark part of the bar shows primary ISA outbreaks. The total number of fish groups transferred to sea each year is indicated on top of each bar.

**Figure 3:**
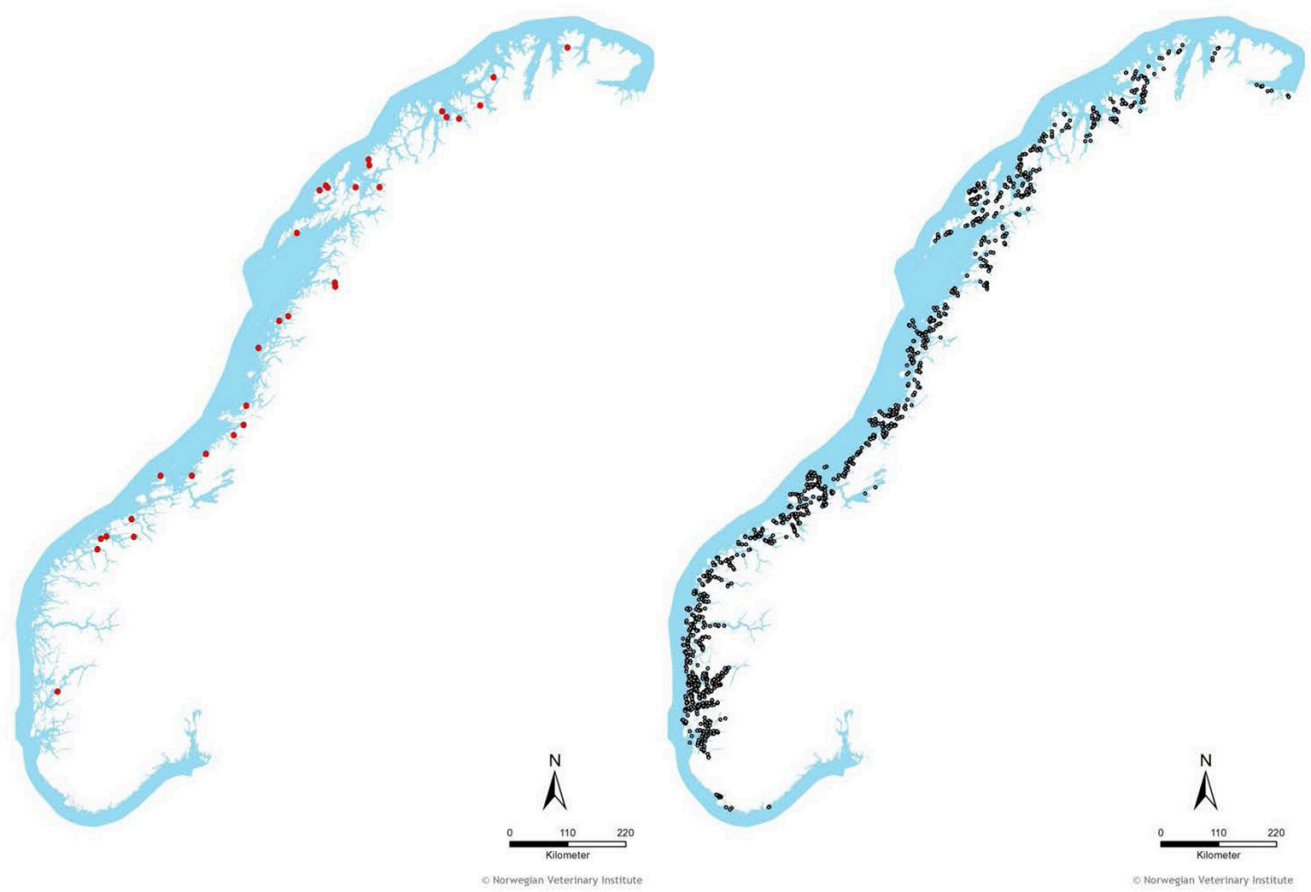
The geographical distribution of fish groups with primary ISA outbreaks (33, red dots, left map) and controls (4438, gray dots, right map) included in the study period (2004–June 2017). A fish group was defined as farmed Atlantic salmon at a given site and separated by fallowing period. Only fish groups stocked for at least 6 months were included in the analysis.

The mean annual probability of having a primary outbreak of ISA in a given fish group was 0.7% (minimum = 0, maximum = 2.1) in the period from 2004 to June 2017. The annual distribution of the number of fish groups with ISA and primary ISA are shown in Figure 2. In total, we identified 4,523 fish groups with farmed Atlantic salmon located on 1,159 unique farm sites during the study period from Jan 2004 to June 2017. Eighty-five of the fish groups, from 81 unique sites, included in our study period were confirmed ISA outbreaks. Out of these, 33 fish groups were identified as primary ISA outbreaks. The remaining 4,438 fish groups were included as controls. Four of the cases were owned by the same company, and were located at two sites, i.e., these sites experienced primary ISA outbreaks twice. This happened in 7% of the 10,000 simulations with random draws. The geographical distribution of cases and controls is shown in Figure 3.

Fish groups that contracted a primary ISA outbreak were on average kept 14 months on-site (minimum 3 and maximum 27) before the ISA outbreak was detected. Primary ISA outbreaks occurred throughout the calendar year, with a peak around spring and late autumn/early winter. The proportion of the first 6 months below a temperature threshold was modeled univariably, with the threshold ranging from 4 to 13 degrees. This variable was only significant with thresholds between 10 and 13, and 12 degrees gave the best model fit. Descriptive statistics, AIC and *p*-values for potential risk factors associated with primary ISA outbreaks are shown in Table 1.

**Table 1:** Descriptive statistics from potential risk factors for control fish groups and fish groups with primary ISA outbreaks.

**Variable**	**Levels**	**Control cohorts**	**Case cohorts**	***p*-value**	**AIC**
Latitude	Mean	63.79	66.85	< 0.001	370.9
	(5%, 95%)	(59.29, 70.07)	(62.56, 70.27)	–	–
	Sd	3.61	3.03	–	–
Log of distance to land	Mean	6.75	6.61	0.19	391.8
(Meters)	(5%, 95%)	(6.05, 7.94)	(6.17, 7.43)	–	–
	Sd	0.6	0.55	–	–
Log of distance to slaughter cage	Mean	3.02	2.83	0.24	392.4
(km)	(5%, 95%)	(1.48, 4.42)	(1.06, 4.28)	-	-
	Sd	0.91	1.1	-	-
Portion of months below 12 degree C	Mean	0.72	0.85	0.004	383.7
(First 6 mo)	(5%, 95%)	(1.85, 17.95)	(1.72, 14.09)	-	-
	Sd	5.13	4.56	-	-
Fallowing duration	Mean	6.78	7.52	0.297	392.7
(Months)	(5%, 95%)	(2, 12)	(2, 12)	–	–
	Sd	4.00	4.39	–	–
Site index	Mean	0.73	0.71	0.453	393.2
	(5%, 95%)	(0.47, 0.89)	(0.53, 0.84)	–	–
	Sd	0.13	0.1	–	–
Transfer to sea	Mean	2010	2009	0.038	389.3
(Year)	(5%, 95%)	(2004, 2016)	(2004, 2014)	–	–
	Sd	3.72	3.3	–	–
Cohort type	Autumn and relocated	2 505	14	0.110	391.15
	Spring	1 933	19	–	–
Stocking duration	1–2 mo	3 453	15	< 0.001	377.6
	>2 mo	985	18	-	-
Weight at sea transfer	Mean	490.14	314.89	0.342	392.42
(Gram per fish)	(5%, 95%)	(70.76, 2149.66)	(68.67, 1393.03)	–	–
	Sd	1075.09	473.27	–	–
Log of max number of fish	Mean	13.31	13.53	0.127	391.1
(First 6 mo)	(5%, 95%)	(11.91, 14.36)	(12.62, 14.19)	–	–
	Sd	0.83	0.61	–	–
Log of max density	Mean	8.76	9.08	0.009	386.8
(First 6 mo)	(5%, 95%)	(7.55, 9.88)	(8.34, 9.96)	-	-
	Sd	0.72	0.72	-	-
Mean mortality	Mean	0.015	0.011	0.400	393.2
(First 6 mo)	(5%, 95%)	(0.001, 0.037)	(0.004, 0.034)	–	–
	Sd	0.013	0.009	–	–
Max mortality	Mean	0.034	0.042	0.417	393.2
(First 6 mo)	(5%, 95%)	(0.002, 0.118)	(0.007, 0.119)	–	–
	Sd	0.057	0.037	–	–
Max APD neighbor eksl. site	Mean	239 466	145 431	< 0.001	377.6
(First 6 mo, numbers in 1000)	(5%, 95%)	(48 255, 545 108)	(33 515, 301 045)	–	–
	Sd	154 258	84 292	–	–
Max APD neighbor incl. site	Mean	251 904	155 480	< 0.001	376.9
(First 6 mo, numbers in 1000)	(5%, 95%)	(59 001, 557 126)	(39 699, 310 106)	–	–
	Sd	155 204	86 556	–	–
HSMI before ISA	No	3 529	25	0.595	393.5
	Yes	909	8	–	–
IPN before ISA	No	3 528	18	< 0.001	383.5
	Yes	910	15	–	–

*The continuous variables were described using mean, 5 and 95% percentiles and the SD. The discrete variables were described with the number of fish groups in each category. P-values and the AIC value based on univariable logistic regression are reported for each variable. The AIC value for a model without explanatory variables was 391.7. ISA, infectious salmon anemia; IPN, infectious pancreatic necrosis; HSMI, heart and skeletal muscle inflammation; APD, area production density; mo, month; max, maximum*.

**Figure 4:**
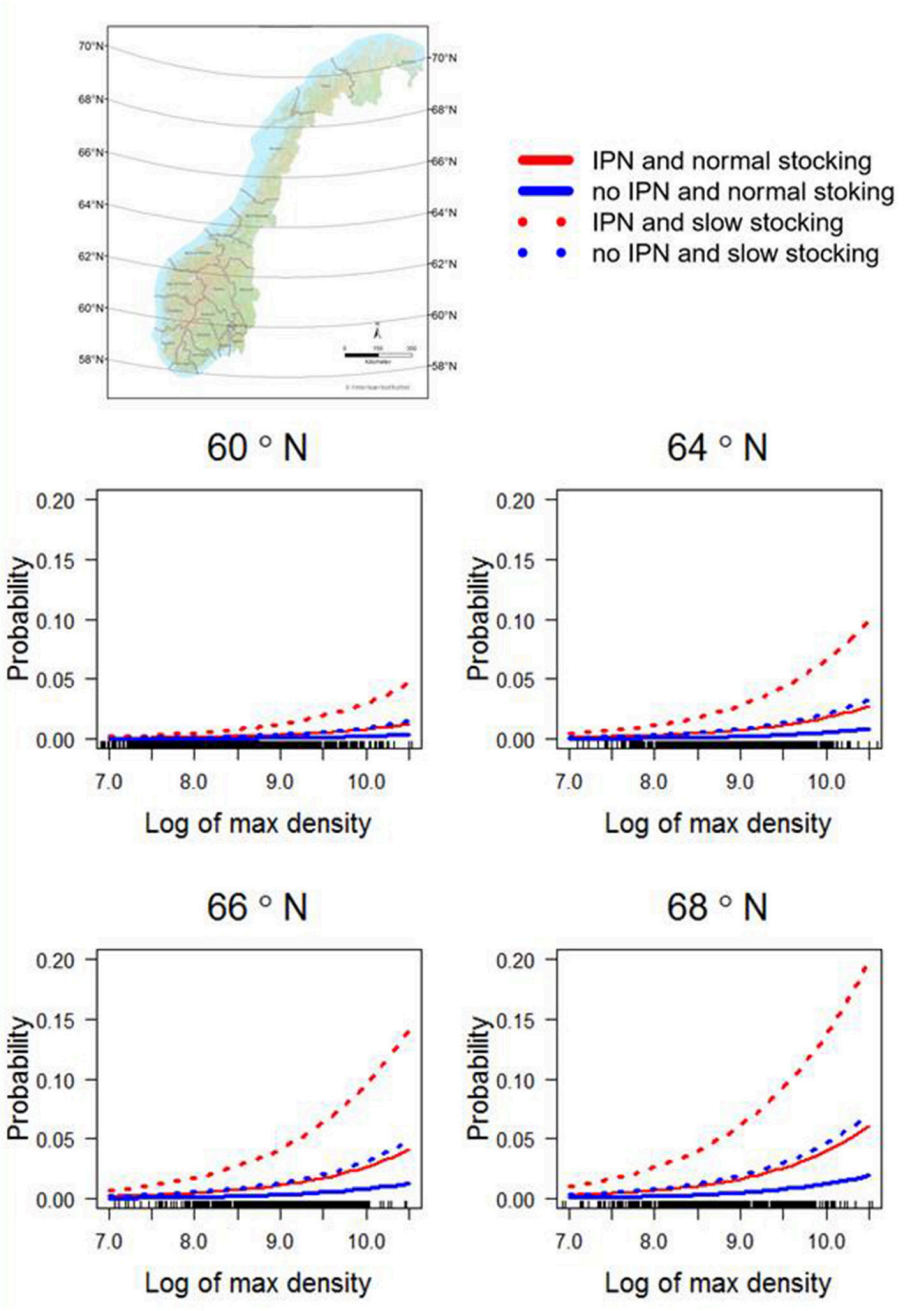
Predictions from the final multivariable model shown for the latitudes 60° N, 64^o^ N, 66^o^ N, and 68^o^ to illustrate how the ISA risk increases with increasing latitude. The latitudes were chosen from areas with considerable salmon production density. For each latitude, density (on x axis), normal (1–2 month; whole lines) or long (≥3 months; dotted lines) stocking period, and the occurrence of infectious pancreatic necrosis 6 months before an outbreak of infectious salmon anemia or during the production period are shown (red if yes, blue if no). The map of Norway shows different latitudes.

In the final multivariable model, the potential risk factors associated with primary ISA outbreaks were (1) the occurrence of IPN, (2) a stocking period longer than 2 months (long stocking), (3) higher latitude, and (4) increased maximum density (log transformed biomass per cage volume) the first 6 months after transfer to the sea site. No interactions were found to be significant or improved the AIC value. Parameter estimates and *p*-values for the final model are shown in Table 2. A sensitivity analysis, excluding 1/20, 1/10, and 1/8 of the controls with highest exposure of ISAV from neighboring farms, gave the same final model with only slightly different parameters: latitude was the most affected parameter, with a maximum increase of 8% in the coefficient (when excluding 1/8 of the controls). A visualization of the model output in four alternative combinations of risk factors are shown in Figure 4.

**Table 2:** Final model selected for the multivariable logistic modeling of risk factors associated with a primary ISA outbreak.

	**Estimate**	**Std. error**	***P*-value**	**OR (95% CI)**
(Intercept)	−27.20	4.66	< 0.001	
Stocking period (>2 mo)	1.36	0.36	< 0.001	3.88 (1.91–7.88)
Latitude	0.21	0.06	< 0.001	
IPN before ISA	1.17	0.37	0.001	3.22 (1.56 – 6.65)
log (max density)	0.89	0.28	0.001	

*Odds ratio (OR) was calculated for the discrete variables. The AIC value for the final model was 348.9*.

## Discussion

The annual probability of having a primary ISA outbreak, i.e., an ISA outbreak with an unknown source of infection, was low during the study period from 2004 to June 2017 (mean annual probability = 0.7%). We identified two subsequent events with primary ISA outbreaks on the same site. The probability of this outcome by pure chance was found to be low, but not unlikely (*p* = 0.07). This result was not significant, but it indicates that properties related to sites might be associated with primary ISA outbreaks.

A possible issue regarding the independence of data used herein, however, may be that some of our presumed primary cases were infected by an unknown undetected neighboring farm infected with HPR-del ISAV, and therefore did not represent a primary ISA outbreak. We argue, however, that this is very unlikely. This assessment is in part supported by the sensitivity analysis; the exclusion of exposed fish groups would alter the model outcome if they included many primary ISA outbreaks. We know that awareness of ISA among farmers and fish health personnel is high, and regular health inspections are carried out by fish health services on all farms at a regular basis. In addition, ISA is a disease that normally causes high mortality (1), and all cases of increased mortality should be reported according to the aquaculture regulations in Norway. See Lyngstad et al. (32) for a further description of these regular health inspections. The real methodological limitations lie in the nature of correlation studies: they do not identify the direction of causality. Variables associated with increased risk, on the other hand, may provide clues of where to look for mechanisms behind primary ISA outbreaks.

### Latitude

The effect of latitude identified herein, i.e., the increased risk of primary ISA outbreaks in Northern Norway during the study period, cannot be explained by data available in this study, and we have not been able to find any clues in the literature. The Pearson correlation between the proportion of first 6 months below 12 degrees Celsius and latitude was 0.52. Using both latitude and temperature as variables in the same model resulted in temperature not being significant. While only using temperature in the final model increased the coefficient for the other variables with between 7 and 10%, the AIC value decreased compared to using latitude. This indicated that latitude partly explains temperature differences between north and south, but environmental drivers, management differences or other unknown factors can also be confounded with latitude. Future research on the geographical structure of salmon aquaculture, management and wild fish might elucidate why northern Norwegian salmon aquaculture farms are more susceptible to primary ISA outbreaks.

### Infectious Pancreatic Necrosis

We found that IPN increased the risk of experiencing a primary ISA outbreak more than two-fold (Table 2), concurrent with previously published results suggesting that fish with a history of IPN are more prone to diseases such as PD and HSMI (33). In contrast to this, another study suggested that IPN provides some protection against the development of ISA (34). It is common to vaccinate salmon against IPN before sea transfer, but vaccination does not fully prevent clinical outbreaks of the disease (35). However, the annual number of IPN outbreaks (including both freshwater and seawater environment) shows a decreasing trend over the last years, probably connected to widespread use of IPN-resistant salmon strains and a targeted effort to eradicate “house strains” of the IPN virus in hatcheries (unpublished work). In 2016 this trend seemed to plateau (36). All these efforts add complexity to the causal relationships behind this finding. A more detailed study of the relationship between ISA and IPN may shed some light on the currently scarce understanding of interactions between different viral infections.

### Stocking

We identified that a long stocking period was associated with an increased risk of contracting a primary ISA outbreak (Table 2 OR = 3.885 and Figure 4). Stocking involves the movement of fish and repeated visits by well-boats; both these factors are associated with an increased risk of infection (23, 37). A likely consequence of a prolonged stocking period is that the well-boats have more intermediate visits to other farms, followed by an increased probability of indirect contact with cohorts infected with both HPR0 ISAV and HPR-del ISAV. A long stocking period may also involve fish from several smolt producers, different size and age groups, and thereby jeopardizes the biosecurity principle of separating the generations.

### Biomass

We found that increasing maximum density during the first 6 months was a significant risk factor, illustrated along the x-axis in Figure 4. High density (biomass per cage volume) is associated with fish abundance, a factor known to affect the susceptibility to both pancreas disease (26) and ISA (12). Risk factors associated with poor management and stress have been associated with ISA outbreaks in early epidemiological studies (21, 22), but these studies did not differentiate between primary or secondary ISA outbreaks.

While the overall risk of contracting a primary ISA outbreak is low, a site with the right combination of risk factors reaches a probability of 0.2, within a reasonable value for maximum density (Figure 4), indicating a possibility of risk reduction through management or risk-based surveillance. Both stocking practices and maximum density can be controlled, and the incidence of IPN can be reduced by choosing IPN-resistant QTL-salmon or vaccination. We do not provide causal relationships between outbreaks and risk factors, however, and the outcome of targeted management is therefore not certain.

## Conclusions

When defining primary ISA outbreaks based on an earlier published transmission model (12), we found the overall probability of having a primary ISA outbreak to be low, but increased with higher latitudes. The occurrence of IPN, long stocking and increasing maximum biomass density increased the probability of getting a primary ISA outbreak. The mechanisms associated with the occurrence of IPN are not fully understood, but we provide clues for further investigation of the subject. Our results indicate that the emergence of primary ISA outbreaks is associated with risk factors that can be managed to some degree, or that may function as guidelines for risk-based surveillance. Further research on understanding the drivers and mechanisms for the emergence of a primary ISA outbreak are needed.

## Author Contributions

TL, LQ, EB, and AK were responsible for planning and performing the study, data analyses, interpretation of results, and co-drafted the manuscript. HS provided the ISAV sequence data used in the calculation of genetic distances. All authors significantly contributed to the revision and editing of the manuscript and approved the final version. All authors read and approved the final manuscript. An earlier version of this work was presented at AquaEpi I 2016, Oslo, Norway as an oral presentation titled Analysis of risk factors for isolated outbreak of infectious salmon anemia (ISA) in marine farms with Atlantic salmon.

### Conflict of Interest Statement

The authors declare that the research was conducted in the absence of any commercial or financial relationships that could be construed as a potential conflict of interest.
